# Snail1-expressing cancer-associated fibroblasts induce lung cancer cell epithelial-mesenchymal transition through miR-33b

**DOI:** 10.18632/oncotarget.23082

**Published:** 2017-12-07

**Authors:** Jia You, Min Li, Yun Tan, Liming Cao, Qihua Gu, Huaping Yang, Chengping Hu

**Affiliations:** ^1^ Department of Respiratory Medicine (Department of Respiratory and Critical Care Medicine), Key Cite of National Clinical Research Center for Respiratory Disease, Xiangya Hospital, Central South University, Changsha, Hunan 410008, P.R. China

**Keywords:** Snail1, cancer-associated fibroblasts, lung cancer, microRNA, epithelial-mesenchymal transition

## Abstract

Lung cancer has a high propensity for metastasis. Cancer-associated fibroblasts (CAFs) are the main type of stromal cells in cancer tissue, are activated by tumor cells, and play a significant role in tumor development. However, whether CAFs induce lung cancer cell metastasis, as well as pathway involved in CAF-induced lung cancer cell metastasis, is uncertain. Snail1 is a transcriptional factor whose expression in the stroma is associated with lower survival rates in patients with cancer. However, how Snail1 regulates the crosstalk between stromal cells and tumor cells when it is expressed in the stroma has not been determined. Altered microRNA (miRNA) expression is correlated with lung cancer metastasis. Our previous study of microRNAs showed that miR-33b levels were clearly reduced in lung cancer cell lines and lung cancer tissues, and miR-33b suppressed tumor cell epithelial-mesenchymal transition (EMT) when its expression was elevated. In this study, we found that co-culturing CAFs with lung cancer cells induced miR-33b downregulation and promoted epithelial cells EMT. Moreover, we found that miR-33b overexpression in lung cancer cells counteracted CAF-induced EMT. Interestingly, Snail1 expression in fibroblasts activate the inductive effects of CAFs on lung cancer cell EMT. Hence, understanding the molecular mechanism underlying the communication between stromal cells and tumor cells mediated by miR-33b may lead to the identification of novel targets for the treatment of lung cancer. Additionally, understanding the role of Snail1 driving CAFs to induce lung cancer cell EMT may provide with a new perspective on the treatment of lung cancer.

## INTRODUCTION

Lung cancer is the most common cause of cancer-related death worldwide, and metastasis is the principal cause of death in patients with lung cancer [1]. Non-small cell lung cancer (NSCLC) accounts for approximately 85% of all lung cancer cases, and its five year survival rate is approximately 15% [2]. Epithelial–mesenchymal transition (EMT) is recognized as one of the main risk factors for metastasis. EMT causes the static apicobasal polarity of epithelial cells to shift to a mesenchymal-like anterior–posterior polarity, which permits the cells to migrate as single units. EMT enables tumor cells to acquire migratory and invasive ability [3]. Single cells generated by EMT migrate from primary tumors, intravasate, extravasate and then relocate to distant organs.

The tumor stroma is believed to exert important effects on cancer cell aggressiveness and drug resistance [4–6]. Cancer-associated fibroblasts (CAFs) are one of the most common cells in the tumor stroma. CAFs promote tumor growth [7], invasion [8] and metastasis [9] by secreting various cytokines. In addition, CAFs secrete a distinctive extracellular matrix (ECM) that facilitates tumor cell attachment and invasion [10]. However, the molecular mechanisms underlying how CAFs induce EMT in lung cancer have not been fully elucidated.

Snail1 is a transcription factor whose function has been widely studied because it initiates EMT [11]. EMT and Snail1 are indispensable for embryonic development, however their contributions to epithelial tumor cell migration remain controversial. Snail1 is normally expressed in a small fraction of cells with fibroblastic morphology that are located in the stroma in breast and colon tumors [12, 13]. Snail1 expression has been reported to be significantly [14, 15] and non-significantly [13, 16] associated with patient survival in breast tumors. Therefore, we evaluated the function of Snail1 in CAFs.

MicroRNAs (miRNA) are endogenous, non-coding RNAs of 18-22 nucleotides in length that post-transcriptionally regulate gene expression [17, 18]. Different miRNAs play different roles in cancer cell proliferation and EMT [19]. Our previous study showed that miR-33b levels were significantly decreased in lung cancer cell lines and lung cancer tissues and that decreased miR-33b expression was associated with lymph node metastasis. Moreover, restoration of miR-33b suppressed tumor cell EMT *in vitro* and *in vivo* [20]. In the present study, we sought to identify the upstream pathway through which miR-33b inhibits cancer cell EMT. We demonstrated that miR-33b plays an essential role in modulating extracellular stimuli and cancer cell behavior. We also investigated whether CAFs induce lung cancer cell EMT and how miR-33b mediates CAF-induced EMT in lung cancer, and whether Snail1 facilitates CAFs function. We sought to identify the functional link among Snail1, CAFs and miR-33b and to unveil the roles of these entities in lung cancer progression.

## RESULTS

### Characterization of primary CAFs and NFs

**Figure 1:**
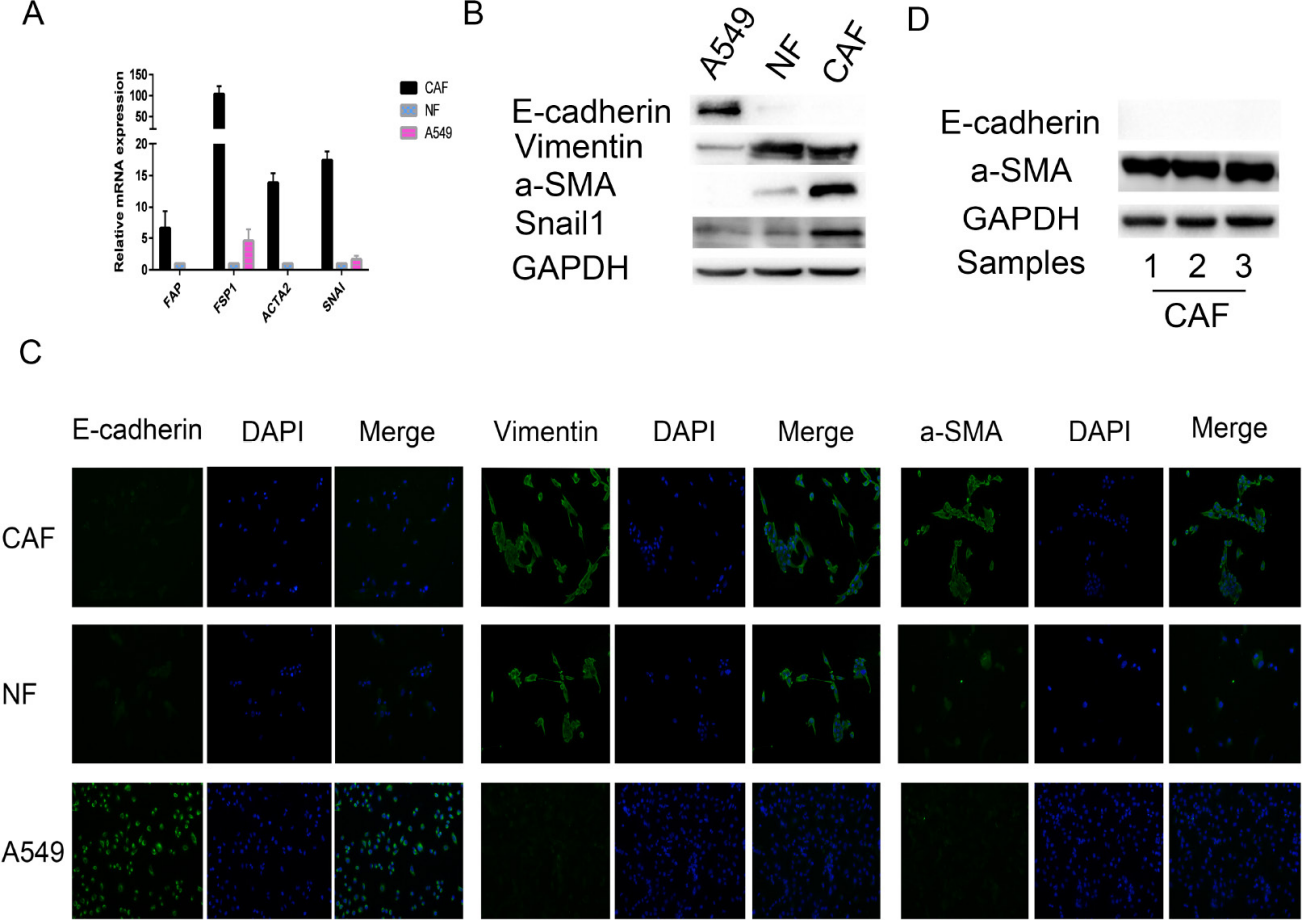
Characterization of primary CAFs and NFs (**A**) The mRNA expression levels of FAP, FSP1, ACTA2, which are CAF specific genes, in A549 cells (an epithelial cell control), NFs and CAFs were detected by qRT-PCR, GAPDH gene was used as the normalization control. We also found that SNAI was overexpressed in CAFs only. (**B**) The protein expression levels of E-cadherin, Vimentin and α -SMA in A549 cells, NFs and CAFs by immunoblotting, using GAPDH protein as the loading control. We also found that Snail1 was overexpressed in CAFs only. (**C**) Immunofluorescence staining revealed the subcellular location and the expression of E-cadherin, Vimentin and α -SMA in A549 cells, NFs and CAFs. (**D**) The expression levels of E-cadherin and a-SMA, which are CAF specific biomarkers, in CAFs isolated from different primary lung cancer tissues were detected by immunobotting. All the primary CAFs showed positive staining for a-SMA and negative staining for E-cadherin, presenting characteristics of CAFs.

CAFs and NFs were separated from three lung cancer tissue specimens and three adjacent normal lung tissue specimens. We examined the expression of fibroblast biomarkers in the CAFs and NFs to determine the purity of the cells. We noted that the expression levels of specific mRNAs, such as fibroblast specific protein 1 (FSP1), fibroblast activation protein (FAP) and alpha-smooth muscle actin (ACTA2), were increased in CAFs, compared with NFs and A549 cells (a control epithelial cell line) (Figure 1A). Western blotting and immunofluorescence assay (Figure 1B, 1C) indicated that: (1) the indicated epithelial cell marker (E-cadherin) was detected only in A549 cells, (2) the indicated mesenchymal cell marker (vimentin) was expressed at a high level in primary cultured fibroblasts (NFs and CAFs), (3) the myofibroblast marker (α-SMA) was expressed at a significantly higher level in CAFs than in NFs and A549 cells, (4) and Snail1 was overexpressed only in CAFs. In particular, CAFs isolated from three different primary lung cancer patiens showed positive staining for the activated myofibroblast marker a-SMA and negative staining for E-cadherin (Figure 1D). Taken together, these results revealed that CAFs of high purity were successfully isolated from the lung cancer tissue specimens.

### Co-culturing lung cancer cells with CAFs induced cell migration and invasion

**Figure 2:**
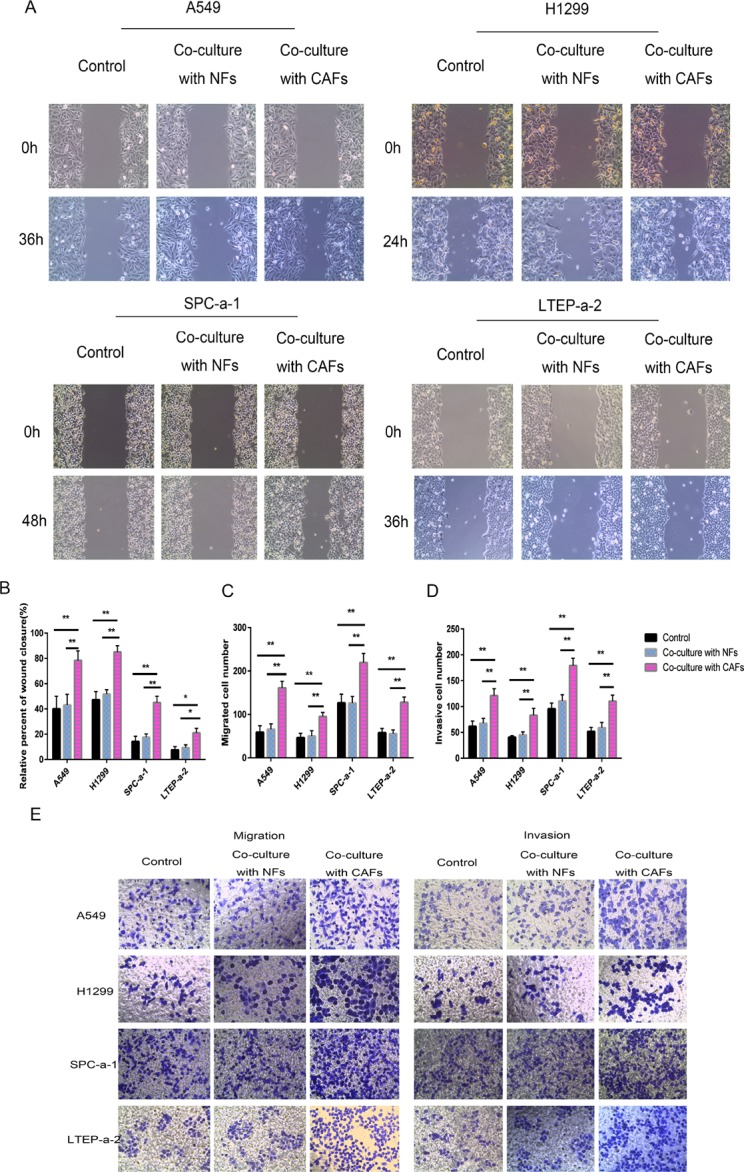
Co-culturing lung cancer cells with CAFs induced cell migration and invasion (**A**, **B**) Cell motility ability was measured by wound-healing assay. The percent of wound closure was determined at 36 h in A549 cells, 24 h in H1299 cells, 48h in SPC-a-1, and 36 h in LTEP-a-2 cells, respectively, before the complete would closure. (**C–E**) Cell migration (C, E) and cell invasion (D, E) were measured by the Transwell cell migration/invasion assay. ^*^P < 0.05, ^**^P < 0.01.

To examine whether CAFs spur lung cancer cell motility, migration and invasion, we co-cultured four different human lung cancer cell lines (A549, H1299, SPC-a-1 and LTEP-a-2) with control medium, NFs or CAFs. Wound-healing assay was conducted to assess whether co-culturing the lung cancer cells with CAFs and NFs affected cancer cell migration rates. As shown in Figure 2A–2B, NFs had no significant effect on cell motility compared with control medium. However, CAFs enhanced cell motility compared with control medium. Transwell assay was performed to evaluate whether CAFs induced cancer cell migration and invasion. The results were consistent with those of wound-healing assay, as the Transwell assay showed that CAFs increased cell migration and invasion in all four lung cancer cell lines (Figure 2C–2E). The results of the abovementioned assays suggest that CAFs secrete factors that act on lung cancer cells to induce migration and invasion.

### CAFs induced miR-33b downregulation and promoted the EMT phenotype in lung cancer cells

**Figure 3:**
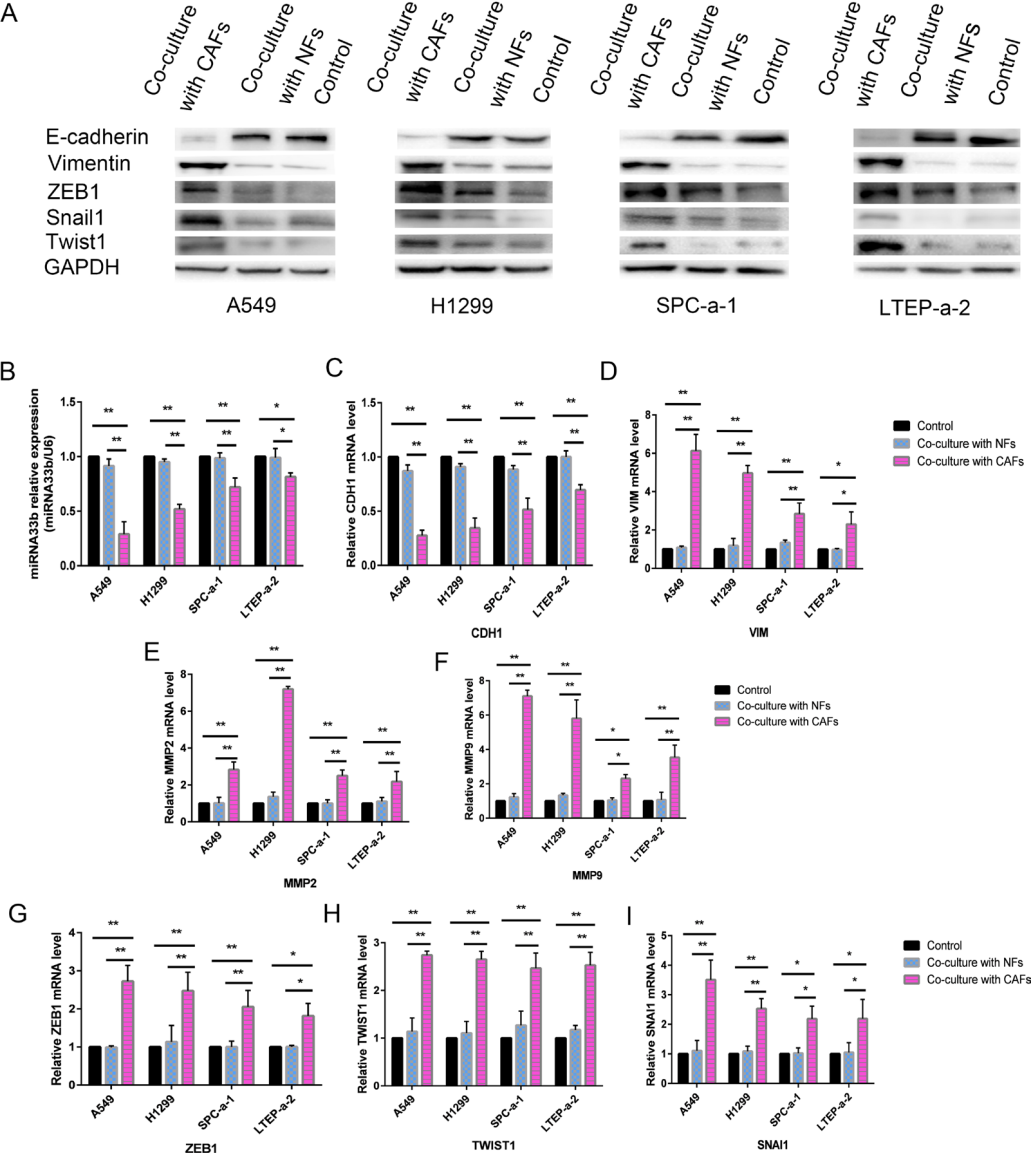
CAFs induced miR-33b downregulation and promoted the EMT phenotype in lung cancer cells (**A**) The protein expression levels of E-cadherin, Vimentin, ZEB1, Twist1 and Snail1 in the lung cancer cells co-cultured with CAFs were deteced by immunoblotting, using GAPDH protein as the loading control. (**B**) The relative level of miR-33b in different NSCLC cell lines co-cultured with CAFs, was detected by qRT-PCR, GAPDH gene was used as the normalization control. (**C–I**) The mRNA expression levels of CDH1 (epithelial marker, encoding E-Cadherin) (C), VIM (mesenchymal markers, encoding vimentin) (D), MMP2 and MMP9 (invasion markers) (E, F), and ZEB1, TWIST1, SNAI1 (EMT-associated transcription factors) (G–I) in different NSCLC cell lines co-cultured with CAFs were detected by qRT-PCR, GAPDH gene was used as the normalization control. ^*^P < 0.05, ^**^P < 0.01.

To determine whether CAFs induced the EMT phenotype in the abovementioned lung cancer cell lines, we measured the levels of the epithelial marker E-cadherin (CDH1), the mesenchymal marker vimentin (VIM), and matrix metalloproteinase 2 (MMP2) and MMP9 in A549, H1299, SPC-a-1 and LTEP-a-2 cells co-cultured with CAFs. The results revealed that E-cadherin expression was decreased, while vimentin expression was increased in A549, H1299, SPC-a-1 and LTEP-a-2 cells co-cultured with CAFs compared with A549, H1299, SPC-a-1 and LTEP-a-2 cells co-cultured with control medium (Figure 3C and 3D). MMP2 and MMP9 expression was increased in cells co-cultured with CAFs compared with cells co-cultured with control medium (Figure 3E and 3F). These results indicated that CAFs induced the EMT phenotype in lung cancer cells. To investigate whether miR-33b was involved in the course of CAFs activating lung cancer cell EMT programming, we examined the expression of miR-33b and its post-transcription factors (EMT-TFs), snail homolog 1 (SNAI1), twist basic helix–loop–helix transcription factor 1 (TWIST1) and zinc-finger E-boxbinding homeobox 1 (ZEB1) in lung cancer cell lines co-cultured with CAFs. We observed that miR-33b expression levels were downregulated, and SNAI1, TWIST1 and ZEB1 expression levels were upregulated in cells co-cultured with CAFs compared with cells co-cultured with control medium (Figure 3B and 3G–3IG). The immunoblotting results confirmed the qRT-PCR assay results (Figure 3A). Taken together, these results revealed that CAFs secrete factors leading to the downregulation of miR-33b in lung cancer cell lines, and then induce the EMT phenotype.

### MiR-33b prevented CAF-induced lung cancer cell EMT

**Figure 4:**
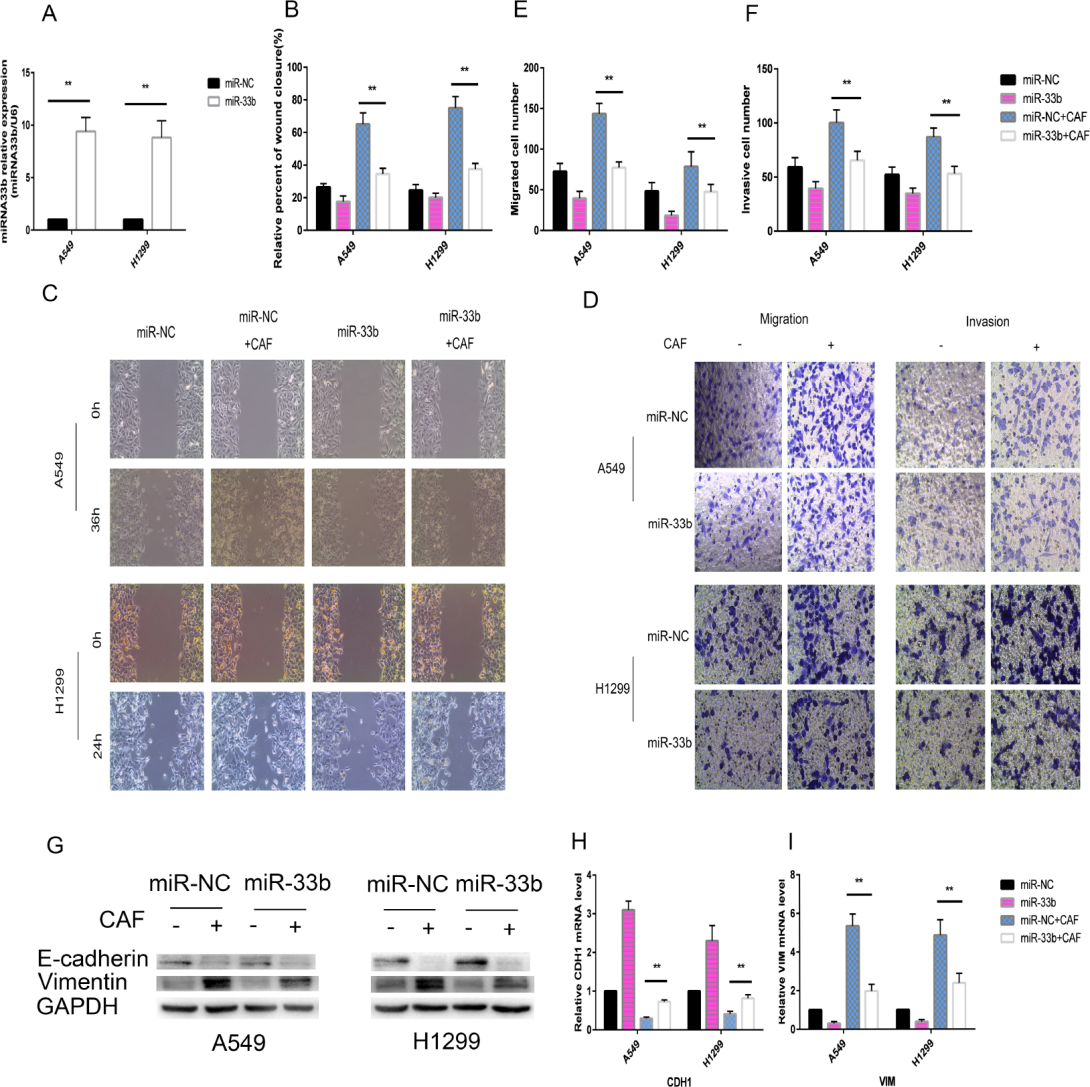
miR-33b prevented CAF-induced lung cancer cell EMT (**A**) A549 and H1299 cells were transfected with miR-33b or miR-NC and then the relative level of miR-33b was measured by qRT-PCR analysis. (**B**, **C**) A549 and H1299 cells were transfected with miR-33b or with miR-NC and, after 48 h, treated or not with CAFs for an additional 48 h. Cell motility was measured by wound-healing assay. (**D–F**) Migration and invasion of A549 and H1299 cells, treated as in (B), was analyzed. (**G**) The levels of E-cadherin and vimentin in A549 and H1299 cells, treated as in (B), were assessed by immunoblotting. (**H**, **I**) The mRNA expression levels of CDH1 and VIM in A549 and H1299 cells, treated as in (B), were assessed by qRT-PCR.

**Figure 5:**
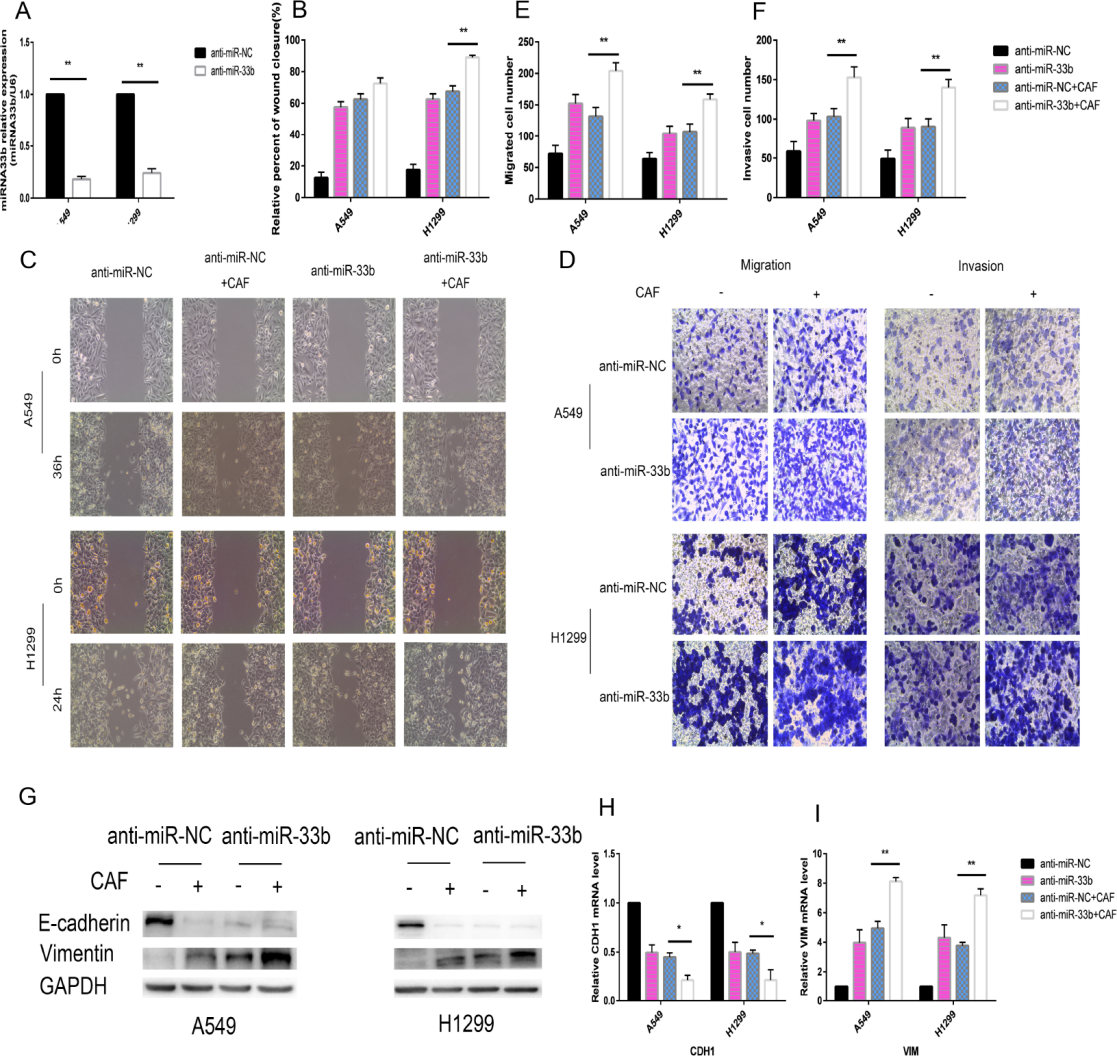
Knockdown of miR-33b promotes CAF-induced EMT of lung cancer cells (**A**) A549 cells and H1299 cells were transfected with anti-miR-33b or anti-miR-NC and then and then the relative level of miR-33b was measured by qRT-PCR analysis. (**B**, **C**) A549 and H1299 cells were transfected with anti-miR-33b or with anti-miR-NC and, after 48 h, treated or not with CAFs for an additional 48 h. Cell motility was measured by wound-healing assay. (**D–F**) Migration and invasion of A549 and H1299 cells, treated as in (B), was analyzed. (**G**) The levels of E-cadherin and vimentin in A549 and H1299 cells, treated as in (B), were assessed by immunoblotting. (**H**, **I**) The mRNA expression levels of CDH1 and VIM in A549 and H1299 cells, treated as in (B), were assessed by qRT-PCR.

To investigate whether miR-33b hinders the malignant interplay between CAFs and lung cancer cells, we increased miR-33b expression levels in A549 and H1299 cells by transfecting them with a miR-33b mimic or miR-NC prior to CAF stimulation. Our data demonstrated that miR-33b was transfected into the above cells with high efficiency and was highly expressed in the infected cells (Figure 4A). When the infected A549 and H1299 cells were stimulated with CAFs, the overexpressed miR-33b efficiently downregulated vimentin expression and prevented the loss of E-cadherin expression in the indicated cells (Figure 4G–4I). The wound-healing assay showed that overexpressed miR-33b counteracted CAF-induced enhancements of lung cancer cell motility rates (Figure 4B, 4C), and Transwell assay showed that miR-33b prevented CAF-induced lung cancer cell migration and invasion (Figure 4D–4F). In contrast, knockdown of miR-33b facilitated CAF-induced lung cancer cell migration, invasion and EMT (Figure 5). Taken together, these data indicate that miR-33b downregulation is a prerequisite for CAF-induced lung cancer cell EMT.

### MiR-33b inhibited CAF-induced lung cancer cell growth and EMT *in vivo*

**Figure 6:**
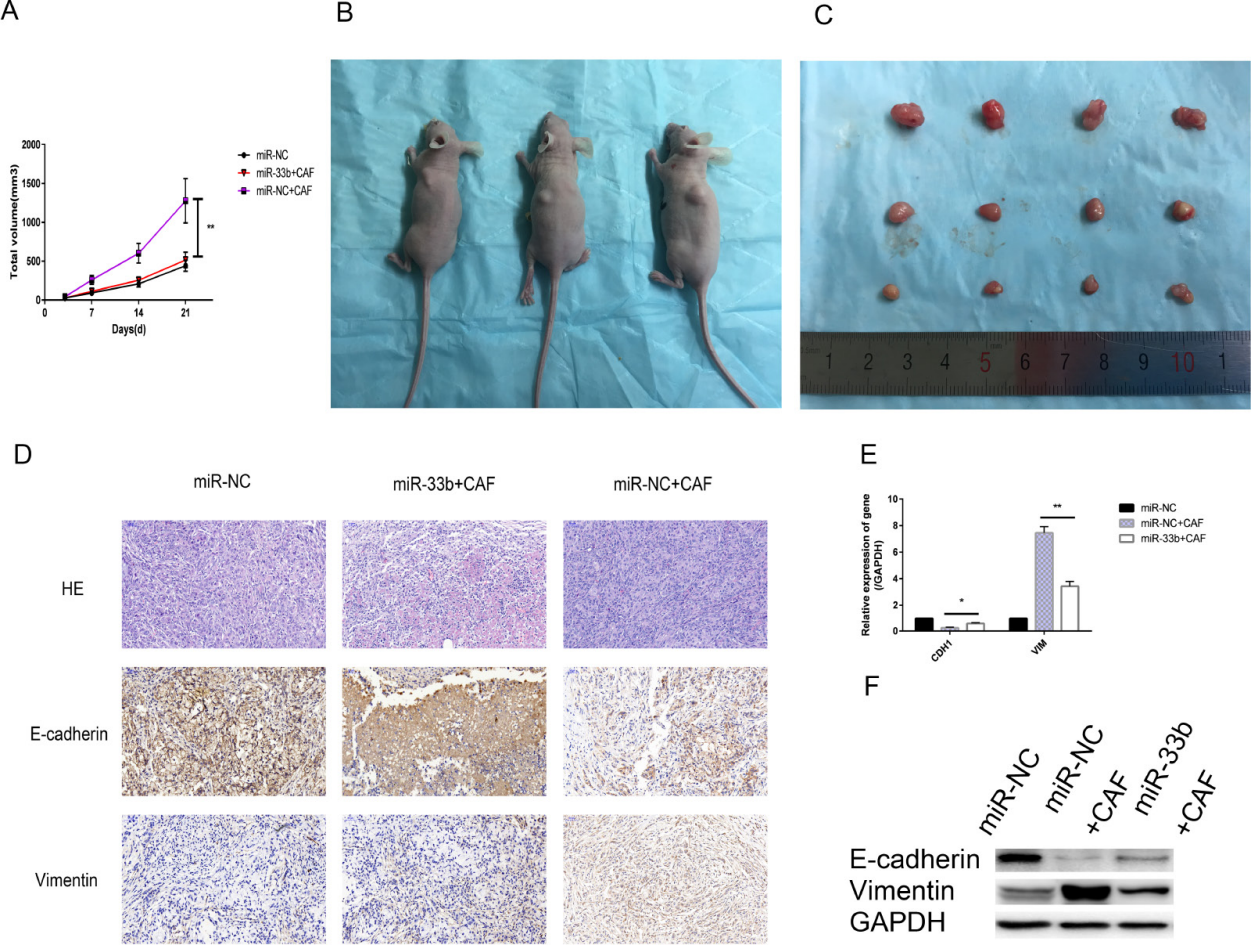
miR-33b inhibited CAF-induced lung cancer cell growth and EMT *in vivo* (**A–C**) Tumor xenograft growth curves in mice (*n* = 6 per group). A549 cells transfected with miR-33b or negative control were inoculated with or without CAFs into nude mice for indicated days. At the experimental end point, tumor xenografts were dissected and photographed. (**D**) Tumor xenografts were processed and stained with HE and immunohistochemically for E-cadherin, vimentin. (**E**, **F**) Levels of vimentin, E-cadherin mRNA and protein in tumor xenografts were assessed by qRT-PCR and western blot analysis. (^*^P < 0.05, ^**^P < 0.01).

To validate the above findings *in vivo*, we analyzed the modulatory effects of miR-33b on tumor growth and lung cancer cell colonization. A549 cells (1 × 10^7^/flank) transfected with lentiviruses carrying miR-33b or a negative control were subcutaneously co-injected with or without CAFs (5 × 10^6^/flank) into the left lateral flanks of nude mice. Xenograft tumors seldom formed when A549 cells were transfected with miR-33b and injected without CAFs into mice. These data showed that the mean volume of the xenograft tumors in mice injected with miR-NC-A549 cells with CAFs was significantly larger than that of the xenografts in mice not injected with CAFs. Additionally, the mean volume of the xenograft tumors in mice injected with miR-33b-A549 cells with CAFs was clearly smaller than that of the xenograft tumors in mice injected with miR-NC-A549 cells with CAFs (Figure 6A–6C). The immunostaining data indicated that the changes in E-cadherin and vimentin expression noted in these experiments were consistent with those noted in the *vitro* experiments (Figure 6D). And the qRT-PCR and western blotting data confirmed the immunohistochemistry data (Figure 6E, 6F). Taken together, these findings demonstrated that miR-33b inhibited CAF-induced tumor growth and EMT *in vivo*.

### Snail1 promoted CAF regulation of lung cancer cells

**Figure 7:**
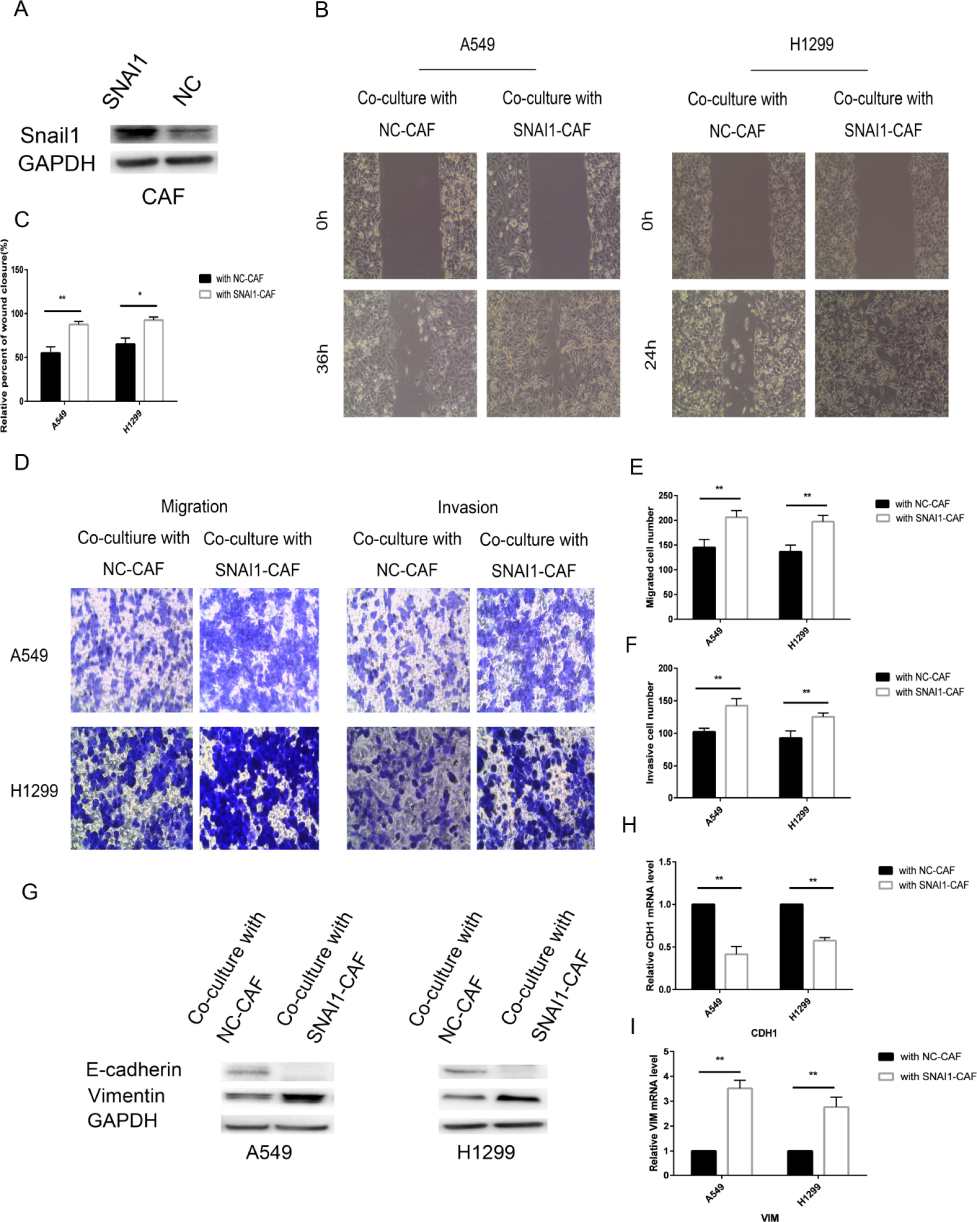
Snail1 promoted CAF regulation of lung cancer cells (**A**) CAFs were transfected with SNAI1 or with negative control and then the expression of Snail1 was detected by immunoblotting analysis. (**B**, **C**) CAFs were transfected with SNAI1 or with negative control and, after 48 h, co-cultured with A549 and H1299 cells for an additional 48 h. Lung cancer cells motility was measured by wound-healing assay. (**D–F**) Migration and invasion of A549 and H1299 cells, treated as in (B), was analyzed. (**G**) The levels of E-cadherin and vimentin in A549 and H1299 cells, treated as in (B), were assessed by immunoblotting. (**H**, **I**) The mRNA expression levels of CDH1 and VIM in A549 and H1299 cells, treated as in (B), were assessed by qRT-PCR.

**Figure 8:**
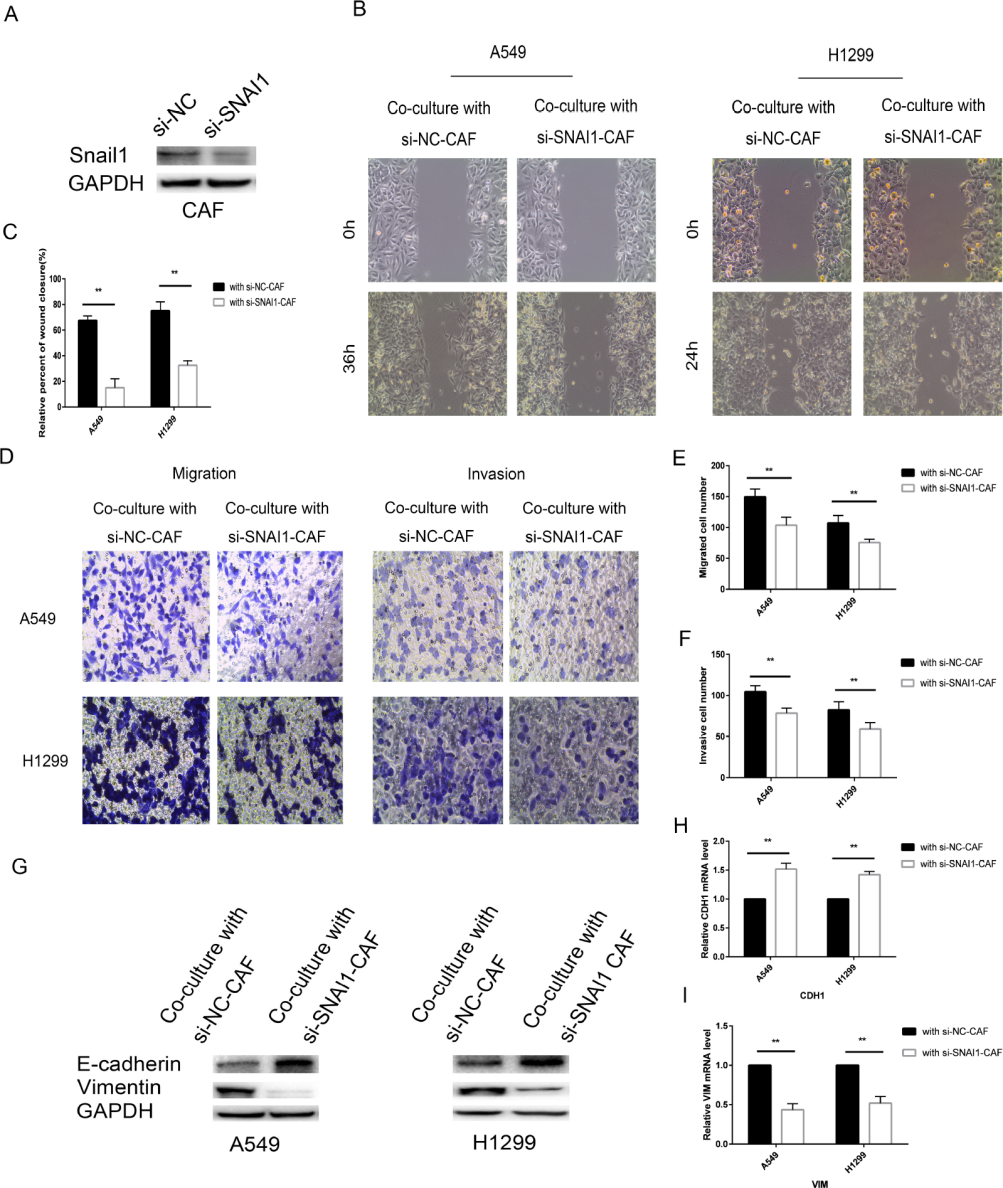
Knockdown of SNAI1 make CAF failed to induce EMT of lung cancer cells (**A**) CAFs were transfected with si-SNAI1 or negative control and then the expression of Snail1 was assessed by immunoblotting analysis. (**B**, **C**) CAFs were transfected with si-SNAI1 or with negative control and, after 48 h, co-cultured with A549 and H1299 cells for an additional 48 h. Lung cancer cells motility was measured by wound-healing assay. (**D–F**) Migration and invasion of A549 and H1299 cells, treated as in (B), was analyzed. (**G**) The levels of E-cadherin and vimentin in A549 and H1299 cells, treated as in (B), were assessed by immunoblotting. (**H**, **I**) The mRNA expression levels of CDH1 and VIM in A549 and H1299 cells, treated as in (B), were assessed by qRT-PCR.

To investigate the role of Snail1 in CAFs, we transfected CAFs with SNAI1 or negative control (NC) or si-SNAI1 or silencing negative control (si-NC). The data showed that SNAI1 was transfected into CAF cells with high efficiency and that SNAI1 was successfully overexpressed in SNAI1-infected CAF cells; however, Snail1 expression was decreased in si-SNAI1-infected CAFs compared with si-NC-infected cells (Figures 7A, 8A). Transfecting CAFs with SNAI1 enhanced the modulatory effects of CAFs on lung cancer cell EMT when CAFs were co-cultured with A549 and H1299 cells, whereas transfecting CAFs with si-SNAI1 attenuated the modulatory effects of CAFs on lung cancer cell EMT (Figure 7G–7I). The expression of E-cadherin, a hallmark of the epithelial phenotype, was decreased, whereas the expression of vimentin, a typical marker of the mesenchymal phenotype, was increased when lung cancer cells were co-cultured with CAFs in which SNAI1 was upregulated. The effect of Snail1 on the modulatory effects of CAFs on cancer cell motility, migration and invasion was confirmed by the wound-healing and Transwell assays (Figure 7B–7F). In contrast, silencing Snail1 expression inhibited CAF-induced lung cancer cell migration, invasion and EMT *in vitro* (Figure 8). Altogether, these findings showed that Snail1 is a critical factor for CAF-induced lung cancer cell EMT.

### Snail1-expressing CAFs induced tumor cell growth and EMT *in vivo*

**Figure 9:**
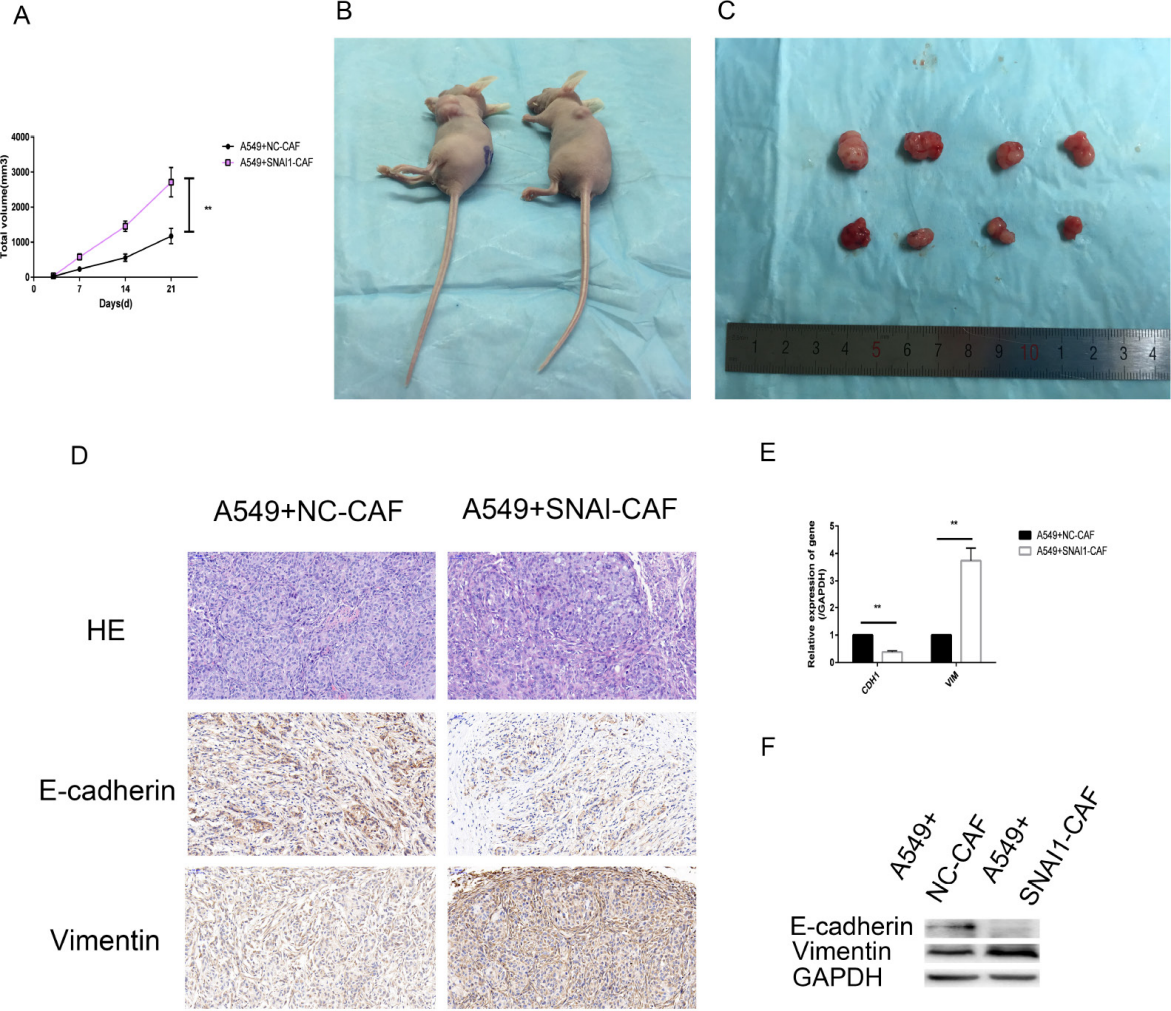
Snail1-expressing CAFs induced tumor cell growth and EMT *in vivo* (**A–C**) Tumor xenograft growth curves in mice (*n* = 6 per group). A549 cells were inoculated with negative control transfected CAFs (NC-CAFs) or Snail1- overexpessed CAFs into nude mice for indicated days. At the experimental end point, tumor xenografts were dissected and photographed. (**D**) Tumor xenografts were processed and stained with HE and immunohistochemically for vimentin, E-cadherin. (**E**, **F**) Levels of E-cadherin, vimentin mRNA and protein in tumor xenografts were detected by qRT-PCR and western blot analysis. (^*^P < 0.05, ^**^P < 0.01).

We also conducted an *in vivo* experiment using xenograft mouse models. A549 cells (1 × 10^7^/flank) were subcutaneously co-injected with negative control-transfected CAFs (NC-CAFs)(5 × 10^6^/flank) or Snail1-overexpressing CAFs (SNAI1-CAFs)(5 × 10^6^/flank) into the left lateral flanks of six nude mice. As shown in Figure 9A–9C, xenograft tumors derived from the co-injection of A549 cells and Snail1-overexpressing CAFs were clearly larger than xenograft tumors derived from the co-injection of A549 cells and NC-CAFs. Immunohistochemical staining, western blotting and qRT-PCR demonstrated that E-cadherin levels were downregulated, and vimentin levels were upregulated when Snail1 was overexpressed in CAFs (Figure 9D–9F). The results of the *in vivo* experiment indicated that overexpressing Snail1 in CAFs reinforced the ability of the cells to support tumor growth and invasion and were consistent with the results of the *in vitro* experiment.

## DISCUSSION

Lung cancer remains the leading cause of cancer-related death worldwide [21], and cancer metastasis is the major cause of death in patients with lung cancer [22]. Epithelial tumor cells leave the primary tumor and metastasis to colonized organs by undergoing EMT [23]. Hence, blocking tumor cell EMT may be a key approach to effectively control cancer metastasis. Emerging evidence supports the idea that the tumor stroma plays a critical role in cancer metastasis [24]. CAFs are a main component of the tumor stroma, which supplies cancer cells with a supportive microenvironment and spurs cancer cell metastatic potential [25–28]. In this research, we demonstrated that CAFs display high a-SMA, FAP and FSP expression and thus exhibit characteristics typical of myofibroblasts [29, 30], however, NFs display very low or undetectable expression of the above proteins. We confirmed that CAFs from patients with NSCLC induced migration and invasion in four lung cancer cell lines with different characteristics. The supportive effects of CAFs on cancer cells have also been demonstrated in prostate cancer, breast cancer and gastric cancer [31–33]. One of the most important factors secreted by CAFs is TGF-β, TGF-β is considered as a major mediator of CAFs activation [34]. Webber *et al*. reported inter-cellular communication between tumor cells and fibroblasts via exosomes expressing TGF-β that lead to CAFs activation [35]. CAFs release interleukins, such as interleukin-6 (IL-6) which promote tumor cells survival and growth [36]. CAFs also secrete factors including VEGF, CXCL12, FGF, IL-8/CXCL8 and PDGF to stimulate tumor angiogenesis [37], CAFs release factors such as HGF [34], CCL5 [38] and stanniocalcin 1 (STC1) [39] to stimulate tumor invasiveness and metastasis. CAFs exposed to gemcitabine increase the release of exosomes which promote recipient epithelial cells drug resistance [40]. Taken together, these findings indicate that the induction of cancer cell EMT by CAFs is a key mechanism underlying the acquisition of aggressive behavior by cancer cells.

The present study was based on our previous study, which focused on the regulatory effects of miR-33b on lung cancer cell EMT [20]. Our previous study showed that miR-33b was downregulated in lung adenocarcinoma cells and that reduced miR-33b expression stimulated ZEB1 expression, which led to WNT/β-catenin signaling pathway activation and thus promoted lung adenocarcinoma progression. We supposed that miR-33b was downregulated by CAFs and consequently promoted cancer cell EMT. Our current data also showed that miR-33b levels were downregulated when cancer cells were co-cultured with CAFs, while miR-33b overexpression was induced by the transfection of cancer cells with miR-33b mimics, resulting in the reversal of CAF-induced EMT. Cirera-Salinas D *et al*. showed that the upregulation of miR-33b expression halted the A549 cell cycle at the G1 phase [41], while a previous study demonstrated that miR-33b inhibited the migration of osteosarcoma cells [42], melanoma cells [43] and breast cancer cells [44].

MiR-33b expression is significantly downregulated in lung cancer cells undergoing CAF-induced EMT. Notably, miR-33b is responsive to signals from the stroma. Researchers revealed that IL-6 secreted from CAFs is higher than NFs, the CM from CAFs promoted EMT and chemotherapy resistance in NSCLC cells through IL-6 signaling [45]. Researchers also showed that CAFs induced EMT in gastric cancer cells via secreting IL-6 that activated JAK2/STAT3 signaling pathway [46]. Andrew M *et al*. revealed that inflammatory response reduced steatosis through activation of hepatic IL-6/STAT3 signaling pathway that subsequently suppressed lipogenic genes (SREBP1c) expression in a fatty liver model [47]. A lot of studies showed that activated STAT3 suppressed SREBP1c promoter activity in hepatocytes [48]. MiR-33b is present in intron 17 of the sterol regulatory element-binding protein 1 (SREBP1) gene on chromosome 17 [49]. Taken together, CAFs may induce lung cancer cell EMT via secreting IL-6 that activate STAT3 and subsequently inhibit miR-33b which locates in SREBP1 gene. However, this pathway may not be the only pathway by which cancer cell EMT is regulated, as the other signals related to CAF-induced cancer cell EMT have not yet been identified. Notably, CAFs can induce lung cancer cell EMT in a miR-33b-mediated manner, suggesting that effective cross-talk occurs between stromal cells and lung cancer cells. Additionally, the study provides novel insight into the molecular mechanism underlying the interplay between CAFs and lung cancer cells. Additional studies are needed to determine which signaling pathways are involved in the effects of miR-33b on lung cancer cell EMT upon CAF stimulation.

In this study, we unveiled a new role for Snail1. We showed that Snail1 is indispensable for the inductive effects of CAFs on epithelial tumor migration and invasion. We co-cultured lung cancer cells with SNAI1-transfected CAFs and showed that the interplay between stromal cells and tumor cells facilitates epithelial tumoral cell EMT. si-SNAI1-transfected CAFs failed to induce epithelial tumoral cell EMT. Lorena *et al*. [50] revealed that Snail1-KO MSCs stopped host fibroblast activation, indicating that Snail1-KO cells prevent tumor invasion by secreting protease inhibitors at higher levels. Studies [51] have shown that the malignant properties (migration, invasiveness and stemness) of tumor cells are consequences of Snail1 expression in cancer cells, which is indirectly promoted by CAFs expressing Snail1. In stroma cells, Snail1-expressing CAFs secrete soluble cytokines and remodel the distinctive architecture of the extracellular matrix (ECM) to maintain a pro-metastatic micro-environment [52, 53]. The rigid ECM and exocrine signaling facilitated by Snail1-expressing CAFs induces Snail1 expression in tumor cells. Snail1 is also a characteristic marker of active fibroblasts and is associated with a poor prognosis, as shown by other researchers [54, 55]. Taken together, these results reveal that Snail1 expression is indispensable for CAF activation. Snail1 expression in epithelial tumor cells is also important, as it promotes breast tumor cell invasiveness [56]. In tumor cells, Snail1 can directly repress the expression of E-cadherin, a gene essential for sustaining the epithelial phenotype, to induce EMT [57]. Altogether, these findings show that Snail1 exerts pro-metastatic effects in epithelial tumors by the following two mechanisms: Snail1 not only promotes total or partial EMT when expressed in epithelial tumor cells, but also favors tumor cell EMT when expressed in stromal cells. We have provided novel insights into the effect of Snail1 on fibroblast-mediated epithelial cell EMT. Snail1 interference may have comprehensive inhibitory effects on tumor metastasis, and Snail1 inhibitors may be useful as a new therapy for epithelial tumors.

Taken together, our findings highlight a role for miR-33b in the suppression of the inductive effects of CAFs on lung cancer cell EMT, however, Snail1 expression in CAFs strengthens the inductive effects of the cells. Therefore, restoration of miR-33b expression and inhibition of Snail1 expression may be useful as novel strategies for treating lung cancer.

## MATERIALS AND METHODS

### Isolation and culture of stromal fibroblasts

The primary cancer tissues used for the isolation of stromal fibroblasts were obtained from three patients with NSCLC who were treated in the Cardiothoracic Surgery Department of Xingya Hospital, Central South University (Hunan, China). The lung cancer tissue specimens were diagnosed as lung adenocarcinoma. The patients did not receive chemotherapy or radiation therapy before surgery. Human lung cancer specimens and adjacent normal tissues specimens that are 3 cm far away from the cancer lesions were collected for this study, which was approved by the Ethics Committee of Xiangya Hospital. Informed consents was obtained from all the patients before they participated in the study.

The fresh tissue specimens were sliced and digested with 160 μg/ml collagenase A (Sigma, St Louis, MO, USA) and 25 μg/ml hyaluronidase (Sigma) for 2 h at 37°C. The cells were then collected and cultured in Dulbecco’s modified Eagle’s medium nutrient mixture F12 (DMEM/F12; Invitrogen, Carlsbad, CA, USA) supplemented with 10% fetal calf serum (FCS; Invitrogen). We passaged the cells when they grew into a monolayer. Homogenous stromal fibroblasts formed after 2–3 passages. All the stromal fibroblasts used in the experiments were from passages less than 10.

### Culture of lung cancer cell lines

The lung cancer cell lines A549, H1299, SPC-α-1and LTEP-α-2 were purchased from the American Type Culture Collection (ATCC; Manassas, VA, USA) and cultured in RPMI-1640 medium supplemented with 10% fetal bovine serum (FBS; Invitrogen, Carlsbad, CA, USA), 100 IU/ml penicillin and 100 IU/ml streptomycin at 37°C in a humid atmosphere with 5% CO2. Cells in the logarithmic phase of growth or of 80% confluence were used for the experiments.

The lung cancer cell lines were co-cultured with stromal fibroblasts using Transwell co-culture insert cells (Costar, New York, NY, USA). Lung cancer cell lines were seeded into lower chamber at a density of approximately 1 × 10^3^ cells/cm^2^, while 2 × 10^3^ cells/cm^2^ fibroblasts into upper chamber, respectively, to reach a ratio of lung cancer cells to fibroblasts of 1:2. It is formed as a non-contacting co-culture system to simulate cancer cell and stromal cell interactions to mimic the tumor microenvironment *in vivo*. The pore diameter of upper chamber is 0.4μm, which is smaller than the size of fibroblasts, which can effectively prevent fibroblasts from crossing the chambers, whereas macromolecules secreted from fibroblasts can easily cross the chambers.

### Wound-healing assay

A total of 5 × 10^5^ cells per well were added to six-well plates to grow into a confluent monolayer. A linear scratch/wound was made in each cell monolayer with a sterile 200 μl pipette, followed by a wash with 1×PBS to remove detached cells. Photomicrographs were taken at 100× magnification, and the distance migrated by the cells was observed at the appropriate time.

### Cell migration and invasion assay

Lung cancer cell migration and invasion abilities was experimented *in vitro* with Matrigel-coated Transwell inserts (BD Biosciences, San Diego, CA, USA). Lung cancer cells were plated in 500 ml serum-free medium in the upper chamber at a density of 5 × 10^4^ cells/well, and medium containing 10% FBS was added to the lower chamber. The cells invaded the Matrigel for the appropriate time, after which the non-invading cells on the upper surface of the membrane were removed with a Q-tip, and the invading cells on the lower surface of the membrane were fixed with methanol and stained using 0.5% crystal violet (Sigma). We counted the numbers of migrating and invading cells in five randomly selected high-power fields with a microscope.

### RNA isolation and quantitative reverse transcription-quantitative PCR (qRT-PCR)

Total RNA was isolated from A549, H1299, SPC-α-1 and LTEP-α-2 cells co-cultured with or without CAFs for 48 h. Total RNA was extracted using TRIzol (Invitrogen, 15596018) according to manufacturer’s instructions. Reverse transcription was performed with a Takara system using random primers. The relative expression levels of target genes were quantified by qRT-PCR with an ABI 7500 StepOne Plus Real Time PCR Instrument (Applied Biosystems, USA) using SYBR Green. qRT-PCR was performed in triplicate for each sample. The 10 μl reaction mixture consisted of template cDNA (0.2 μl), primers (0.4 μl, l.0 M), ROX Reference Dye II (0.2 μl), dH2O (4.2 μl) and SYBR Premix Ex Taq (5 μl, SYBR Premix Ex Taq Kit, Takara). The sequences of the primers used in our study are listed in Table 1. The relative expression levels of the target genes were normalized to those of GAPDH, which was used as an internal control. The ΔΔCt values (co-cultured with CAFs versus without CAFs) are expressed as fold changes.

**Table 1:** Study primers

Genes	Primers 5′–3′
**Human**	
GAPDH	F: 5′-AGGGCTGCTTTTAACTCTGGT-3′
	R: 5′-CCCCACTTGATTTTGGAGGGA-3
CDH1	F: 5′-GGGTTATTCCTCCCATCAGC-3′
	R: 5′-GTCACCTTCAGCCATCCTGT-3′
VIM	F: 5′-GTACCGGAGACAGGTGCAGT-3′
	R: 5′-AACGGCAAAGTTCTCTTCCA-3′
ZEB1	F: 5′-CTGCCCAGTTACCCACAATC-3′
	R: 5′-CAGGGCTGACCGTAGTTGAG-3′
TWIST1	F: 5′-GAGCAAGATTCAGACCCTCAAG-3′
	R: 5′-CCATCCTCCAGACCGAGAAG-3′
SNAI	F:5′-TGCGTCTGCGGAACCTG-3′
	R:5′-GGACTCTTGGTGCTTGTGGA-3′
**Mouse**	
GAPDH	F: 5′-AACTTTGGCATTGTGGAAGG-3′
	R: 5′-TGTGAGGGAGATGCTCAGTG-3′
CDH1	F: 5′-TCTCTTGTCCCTTCCACAGC-3′
	R: 5′-TTCCTGACCCACACCAAAGT-3′
VIM	F: 5′-CGCAGCCTCTATTCCTCATC-3′
	R: 5′-AGCGAGAAGTCCACCGAGT-3′
ZEB1	F: 5′-GTGTGCCTGAACCTCAAACC-3′
	R: 5′-AGCCTCCTGTAACCTGCTGA-3′
TWIST1	F: 5′-TGAGCAAGATTCAGACCCTCA-3′
	R: 5′-CAGCTTGCCATCTTGGAGTC-3′
SNAI	F: 5′-CGTGTGTGGAGTTCACCTTC-3′
	R: 5′-CCAGGAGAGAGTCCCAGATG-3′

F, forward; R, reverse.

### Immunoblotting

The cells were lysed using RIPA buffer containing phosphatase inhibitor cocktail I (Sigma) and a protease inhibitor cocktail mini-tablet (Roche Diagnostics, Indianapolis, IN). The proteins in the lysates (20 μg) were separated by SDS-PAGE and transferred onto a polyvinylidene difluoride (PVDF) membrane (Millipore, Billerica, MA, USA). After the membrane was blocked with 5% non-fat milk in TBST, it was incubated with the following primary antibodies over night at 4°C : E-cadherin (ab76055, 1:1,000; Abcam, Cambridge, MA, USA), vimentin (D21H3, 1:1,000; Cell Signaling Technology, Danvers, MA, USA), ZEB1 (NBP2-23484; 1:800, Novus Biologicals, Littleton, CO, USA), Twist1 (ab175430, 1:1000, Abcam), Snail1(C15D3, 1:1,000; Cell Signaling Technology, Danvers, MA, USA), a-SMA (ab5694, 1:1,000; Abcam, Cambridge, MA, USA) and GAPDH (14C10, 1:1,000; Cell Signaling Technology). The membrane was then washed three times with TBST and incubated with horseradish peroxidase (HRP)-conjugated secondary goat anti-rabbit and goat anti-mouse (1:5,000) immunoglobulin G (Invitrogen). The western blots were visualized using the enhanced chemiluminescence reagents (Millipore, WBKLS0100), and the western blotting results were semi-quantified using ImageJ software (NIH, USA).

### Immunofluorescence

The cells were washed twice with phosphate-buffered saline (PBS), fixed with 4% paraformaldehyde, and then permeabilized with 0.1%Triton X-100 for 10 min. The cells were subsequently blocked with 1% BSA for 1 h at room temperature before being incubated with primary antibodies against a-SMA, vimentin and E-cadherin (1:100 dilution) over night at 4°C. The cells were then washed and incubated with the appropriate secondary antibodies in PBS (1:100 dilution) for 1 h. The cell nuclei were stained with DAPI for 10 min at room temperature. Photomicrographs were taken with a microscope (Leica Microsystems, Heidelberg, Germany).

### Immunohistochemistry

The tissue specimens were fixed with formalin and embedded in paraffin. Immunohistochemical staining was performed using the following primary antibodies: anti-a-SMA, anti-Vimentin and anti-E-cadherin. The appropriate secondary antibodies were used at the appropriate concentrations. Staining was performed with diaminobenzidine, and counterstaining was performed with hematoxylin.

### Gene expression vector construction, lentivirus production and cell transfection

Overexpressed RNAs and Small interfering RNAs (siRNAs) specific for Snail1, as well as miR-33b mimic and anti-miR-33b, were synthesized by Shanghai GenePharma Co., Ltd. (Shanghai, China). Unrelated sequences were used as negative controls and were provided by Shanghai GenePharma (Table 2). Lentiviral vector was used to over express or silence miR-33b or SNAI1 expression in cells. (Shanghai Genechem Co., Ltd., Shanghai, China). The sequences are listed in Table 3. Each recombinant lentivirus was purified by ultracentrifugation, and the titers of these viral particles were expressed as multiplicities of infection (MOIs). The vectors containing the miR-33b mimic and miRNA mimic NC were designated as pGC-FU-miR-33b and pGC-FU-NC-LV, respectively, the vectors containing the anti-miR-33b and negative control were designated as pFU-GW-miR-33b and pFU-GW-RANI-NC, respectively, the vectors containing the SNAI1 and negative control were designated as LVKL19911-1 and LVKL- NC, respectively, and the vectors containing the si-SNAI1 and negative control were designated as LVpFU-GW-007 and LVpFU-GW-NC, respectively. The cells were grown overnight and then transfected with the different lentiviruses at different MOIs (20 for the A549 cell line, 2 for the H1299 cell line and 20 for the CAFs) for 12h, after which the culture medium was replaced. The cells were then collected for different assays.

**Table 2:** RNA oligo sequences

Oligo	Oligo sequences
Snail1-siRNA	Sense: 5′-CCAAGGATCTCCAGGCTCGAA-3′
	Antisense: 5′-TTCGAGCCTGGAGATCCTTGG-3′
siRNA-NC	Sense: 5′-UUCUCCGAACGUGUCAGCUTT-3′
	Antisense: 5′-ACGUGACACGUUCGGAGAATT-3′
miR-33b mimic	Sense: 5′-GUGCAUUGCUGUUGCAUUGC-3′
	Antisense: 5′-AAUGCAACAGCAAUGCACUU-3′
miRNA mimic NC	Sense: 5′-UUCUCCGAACGUGUCAGCUTT-3′
	Antisense: 5′-ACGUGACACGUUCGGAGAATT-3′

**Table 3:** Lentiviral sequences

Lentiviral	Lentiviral sequences
Pri-miR-33b	Forward:5′-GGATCCCTTTGGAGGCCCTGCATCAGGAGGGCTGGACAGCTGCTCCCGGGCCGGTGGCGGGTGTGGGGGCCGAGAGAGGCGGGCGGCCCCGCGGTGCATTGCTGTTGCATTGCACGTGTGTGAGGCGGGTGCAGTGCCTCGGCAGTGCAGCCCGGAGCCGGCCCCTGGCACCACGGGCCCCCATCCTGCCCCTCCCAGAGCTGGAGCCCTGGTGACCCCTGCCCTGCCTGCCACCCCCAGGCCGTGCAGCTGTTCCTGTGTGACCTGC-3′
	Reverse:5′-GCAGGTCACACAGGAACAGCTGCACGGCCTGGGGGTGGCAGGCAGGGCAGGGGTCACAGGGCTCCAGCTCTGGGAGGGGCAGGATGGGGGCCCGTGGTGCCAGGGGCCGGCTCCGGGCTGCACTGCCGAGGCACTGCACCCGCCTCACACACGTGCAATGCAACAGCAATGCACCGCGGGGCCGCCCGCCTCTCTCGGCCCCCACACCCGCCACCGGCCCGGGAGCAGCTGTCCAGCCCTCCTGATGCAGGGCCTCCAAAGGGATCC-3′
Anti-miR-33b	Forward: 5′-GTGCATTGCTGTTGCATTGC-3′
	Reverse: 5′-GCAATGCAACAGCAATGCAC-3′
Anti-miR-NC	Forward: 5′-TTCTCCGAACGTGTCACGT-3′
	Reverse: 5′-ACGTGACACGTTCGGAGAA-3′

### Animal experiments

The *in vivo* experiments were approved by the Animal Center of Central South University (Hunan, China) and were conducted in accordance with the guidelines for the care and maintenance of laboratory animals. A total of 1 × 10^7^ A549 cells that were transfected with lentiviruses carrying miR-33b or negative controls for 36 h were subcutaneously co-injected with or without 0.5 × 10^6^ CAFs in a total volume of 0.2ml of PBS into the left lateral flanks of female BALB/c nude mice (aged 4 weeks). The animals (six per group) were monitored, and tumor size was measured by a caliper. Tumor volumes were determined by measuring the length (L) and the width (W) and then using the following equation: V = (LW2)/2. The mice were euthanized on day 21. The excised tumors were photographed, weighed and sectioned for hematoxylin and eosin (H&E) staining, immunohistochemistry, qRT-PCR and western blotting.

### Statistical analysis

The data are presented as the mean ± standard deviation. Student’s *t*-test was used to analyze the differences between the experimental group and the control group. *P* < 0.05 was considered statistically significant.
